# The Spatial and Heterogeneity Impacts of Population Urbanization on Fine Particulate (PM_2.5_) in the Yangtze River Economic Belt, China

**DOI:** 10.3390/ijerph16061058

**Published:** 2019-03-23

**Authors:** Weiwei Xie, Hongbing Deng, Zhaohui Chong

**Affiliations:** 1 School of Economics and Management, China University of Geosciences, 388 Lumo Road, Wuhan 430074, China; xieweiwei@cug.edu.cn (W.X.); denghongbing_2005@126.com (H.D.); 2 Business School, Shantou University, 243 Daxue Road, Shantou 528400, China

**Keywords:** population urbanization, PM_2.5_, heterogeneity effect, spatial econometric, the Yangtze River Economic Belt

## Abstract

This paper addresses the effect of population urbanization on Fine Particulate (PM_2.5_) in the Yangtze River Economic Belt in China from 2006 to 2016 by employing PM2.5 remote sensing data and using the Stochastic Impacts by Regression on Population, Affluence and Technology (STIRPAT) model. The study contributes to the growing empirical literature by addressing heterogeneity, spillover, and dynamic effects in the dynamic spatial panel modeling process simultaneously. The empirical results show that population urbanization has a significant impact on PM_2.5_ with a positive spillover effect and a dynamic effect being detected and controlled. The heterogeneity effects of population urbanization on PM_2.5_ due to geographical positions show evidence of an obvious inverted U-shaped curve relationship in the upstream area and an increasing function curve in the midstream and downstream areas. The heterogeneity effects due to population urbanization levels show that an inverted N-shape curve relationship exists in low and medium urbanization level areas, while a U-shape curve relationship exists in high urbanization level areas. It is hoped that this study will inform the local governments about the heterogeneity of population urbanization and spillover effects of air pollution when addressing air pollution control.

## 1. Introduction

During the past four decades, urbanization in China has increased rapidly from 17.92% in 1978 to 58.52% in 2017. This rapid urbanization is being accompanied by the agglomeration of the urban population, the utilization of urban land, and severe industrial emissions leading to high ambient air pollution [1,2]. The standardized daily value of PM_2.5_ in China is 75 μg/m^3^, meaning when the daily average concentration of PM2.5 higher than 75 μg/m^3^, the air quality reaches the level of pollution, which is three times that of the World Health Organization (WHO) standard (25 μg/m^3^) [3]. According to the air quality monitoring results of 388 Chinese cities in 2016, only 84 cities reached the air quality standard, accounting for 24.9% of the cities tested. Of the tested cities, 254 cities, that is, approximately 75.1%, did not reach the standard.

The relationship between urbanization and air pollution has become a crucial issue for governments, residents, and academics [4]. Regarding this, many extant studies refer to the Environment Kuznets Curve (EKC) hypothesis analysis framework. In their classic work, Grossman and Krueger (1991) tested the EKC hypothesis and found that per capita income exhibits an inverted U-shaped curve relationship on waste emissions [5]. That is, the effect on the environment may worsen before it gets better as per capita income grows. 

The EKC hypothesis was also utilized to detect the relationship between urbanization and the environment [6,7,8]. However, despite the plethora of studies that has examined the influence of rapid urbanization using various empirical methods and datasets on different spatial scales, the findings have been inconsistent. Ji et al. examined the impacts of urbanization on PM_2.5_ based on 79 developing countries from 2001 to 2010 and found an inverted U-shaped curve, in which low urbanization level countries have an increase tendency while high urbanization level countries have a decrease tendency [9]. Contrary to these findings, Xu et al. [10] used an extended STIRPAT model from 2005 to 2015 to explore the heterogeneity effect between urbanization and air pollutants based on provincial panel data in China. They pointed out there were different relationship curves of PM_2.5_ and population urbanization in different regions. There was a U-shaped curve in the eastern region, a linear relationship in the central region, and a U-shaped or inversed N-shaped curve in the western region of China [10]. Other scholars such as Lee and Oh obtained findings pointing to a similar U-shaped curve relationship between the environment and urbanization [11]. Additionally, other types of relationships between urbanization and pollution were proposed, such as inverse N-shape curve and N-shaped curve [12,13,14].

Based on the above, this study focuses on the following questions.

First, the heterogeneity impact of urbanization on PM_2.5_ is one of the research questions of this study. Heterogeneity includes many aspects. Ji and Chen studied the heterogeneity of urbanization impact on energy consumption analyzed in different stages of urbanization and the heterogeneity of the energy-saving effect of urbanization at different income levels [15]. Sun et al. considered urban traffic infrastructure investment in air pollution due to regional heterogeneity and city-scale heterogeneity [16]. In our study, we take regional heterogeneity and urbanization heterogeneity into consideration. The regional heterogeneity of PM_2.5_ may be correlated to different geographical conditions and urbanization levels. Considering China’s large geographical area and complex terrain, the influence of urbanization on PM_2.5_ in regions with dissimilar urbanization levels may be significantly different. Most of the urban areas in East and Central China exhibited population density increases along with PM_2.5_ decreases, while other areas in China show opposite tendencies [17]. Due to the significant differences in socioeconomic development, complex terrain and landforms, a regional heterogeneity effect of urbanization on air pollution should be investigated and compared [18]. Although many previous studies have focused on the heterogeneous influence of geographical characteristics [8,19,20], the difference in the urbanization level has been neglected. However, the associations between urbanization and PM_2.5_ may show heterogeneous patterns in the regions with different geographical characteristics and urbanization levels. 

Second, the lack of consideration of dynamic effects and spatial dependence may also lead to a problematic outcome, causing estimation bias and leading to unreliable results. Wu et al. utilized a dynamic panel model to confirm the EKC hypothesis in China as a whole, as well as in East, Central, and West China separately [21]. The fixed effect results show that a significantly negative effect of population urbanization on PM_2.5_ appears in West China, and insignificant effcts in East and Central. However, when the model takes the time-lag effect into consideration, the result of the dynamic system generalized method of moments (GMM) indicated that population urbanization has a positive impact on PM_2.5_ in West China and a significantly negative relationship in Central China. The spatial spillover effect of urbanization on PM_2.5_ means urbanization in one area influence PM_2.5_ in another adjacent area. In Du et al., the spillover effect was explained as the influence of neighboring urbanization in terms of the spatial dependence of PM_2.5_ concentrations [20]. They explored the spillover effect of urbanization on PM_2.5_ in the Beijing–Tianjin–Hebei region, the Pearl River Delta, and the Yangtze River Delta from 2000 to 2010 with a spatial lag model, and claimed that urbanization had different spatial direct and indirect effects on PM_2.5_. To our knowledge, few studies have considered these two problems simultaneously.

Third, each city has its own urbanization process, and therefore using city level data is superior to provincial data. Existing studies have focused mainly on rural areas or provinces, and few have investigated prefecture-level cities [21,22,23]. The research of prefecture-level city could provide more detailed information, which help to stimulate a more accurate estimation, whereas national or provincial data may show bias [24]. Furthermore, previous literature includes mainly cross-sectional data, ignoring the characteristic of dynamic changes concerning the effect of urbanization on air pollution [25,26]. 

Fourth, many studies addressing PM_2.5_ concerntration in China are based on data from air quality monitoring stations, which have time limitations. As in China, full coverage of monitoring stations in urban area were built to detect PM_2.5_ and other atmospheric pollutants after 2013. As a result, the existing studies in China usually have a tendency to focus on PM_2.5_ concentrations for short-time series, such as daily, monthly, and single yearly. Wang and Fang used PM_2.5_ data obtained from 241 new observation points in 54 cities of the Bohai Rim Urban Agglomeration in 2014 to determine the spatial–temporal distribution of PM_2.5_ and its socioeconomic determinants [27]. Guo et al. used the daily PM_2.5_ of 35 monitoring sites from April 2013 to March 2015 that were calculated from daily Air Quality Index data and were collected from the official website of the Beijing Municipal Environmental Protection Bureau [28]. Wang et al. employed the data from monitoring stations to analyze the spatial distribution of PM_2.5_ in 190 cities in China in 2014 [29]. However, short-time data cannot reflect stage heterogeneity. Additionaly, the monitoring stations are point measurements and it is not clear to represente the air quality of a given area. Fortunately, remote sensing data we used in the study helps to overcome the two drawbacks above.

Consequently, this study focuses on the following research highlights. (1) We use prefecture-level data to discuss the heterogeneity impact of population urbanization on PM_2.5_ from two perspectives: regional heterogeneity and urbanization heterogeneity. To address regional heterogeneity, we divide the Yangtze River Economic Belt (YREB) into three parts: upstream, midstream, and downstream. For the urbanization heterogeneity, the data sample is divided into three categories based on the annual growth rate of urbanization from 2006 to 2016: low, medium, and high urbanization levels. (2) To avoid possible estimation bias caused by spatial interaction effects and the dynamic effect, we address these two issues using a combination of a dynamic model and a spatial econometric specification. We believe that this integrated modeling framework provides new insights on the relationship between population urbanization and PM_2.5_ in China. (3) Panel data from prefecture-level cities are used for our study. The data concerning PM_2.5_ are from a combination of Aerosol Optical Depth retrievals from multiple satellite instruments.

This study is organized as follows. The next section describes the study area, dataset, and the empirical methods, while Section 3 presents the empirical results. The final section discusses the dynamic, spillover, and heterogeneity effects, respectively, and then presents the conclusions.

## 2. Study area and Method 

### 2.1. Study Area

**Figure 1:**
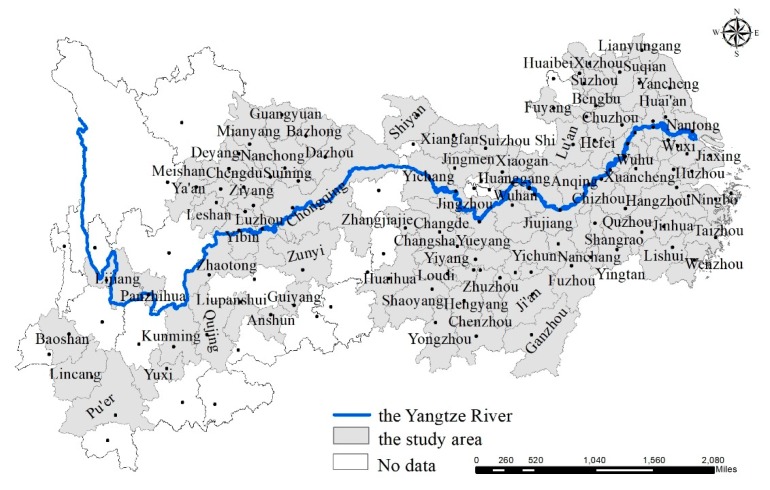
Study area.

The YREB is the biggest economic belt in China, covering nine provinces (Jiangsu, Zhejiang, Anhui, Hubei, Hunan, Jiangxi, Sichuan, Guizhou, and Yunnan) and two municipalities governed directly by the central government (Shanghai and Chongqing). The YREB stretches across the eastern, central, and western regions of China, accounting for over 20% of China’s geographical area (Figure 1). 

In 2011, the construction of an ecological civilization—especially the green development of the YREB—became a national strategy. “The outline of Yangtze River Economic Belt Development Plan” formally became a national development strategy in 2016 aiming to promote new urbanization for the YREB development [30]. This strategy would provide new opportunities for further development of the cities in the Yangtze River basin and promote urbanization in China. In 2016, the YREB was home to more than 40% of China’s population and contributed a similar percentage of the gross domestic product (GDP). Then, the proportion of urban population in the YREB increased from 42.4% in 2006 to 56.9% in 2016. However, most cities in the YREB are facing more serious air pollution than other cities in China, especially the cities in the Yangtze River Delta, which have become a focus of public concern [29].

### 2.2. Empirical Method

#### 2.2.1. STIRPAT Model

The STIRPAT (Stochastic Impacts by Regression on Population, Affluence and Technology) model is a classic theoretical framework for PM_2.5_ research developed on the basis of the IPAT model [31]. In the early 1980s, Ehrlich and Holdren pioneered the Influence, Population, Affluence, and Technology (*IPAT*) model (*I* = *PAT*) to analyze the environmental effects of population and economic variables. In the equation, *I* represents the environmental impact, *P* represents the population, A refers to affluence, and *T* refers to technology. The *IPAT* model is widely recognized and applied for its simple and effective analysis of environmental impact. However, the model has two drawbacks: First, the *IPAT* model is a purely mathematical model which cannot be tested directly by empirical data, and the hypothesis of various factors affecting the environment cannot be obtained [32]. Second, the *IPAT* model simply assumes that the elasticity of population, wealth, technology, and environment are unified. Due to these limitations, Dietz and Rosa improved it to the STIRPAT model. It allows other explanatory and control variables to be added, which is more flexible [32,33]. The STIRPAT model is currently widely used to analyze the environmental impact of population and economic factors [34,35,36]. The expression of STIRPAT can be seen in Equation (1).

(1) $$I=\alpha P^{\beta }A^{\gamma }T^{\lambda }\mu$$

where *I*, *P*, and *A* have the same meanings as in the model; α is the constant term; β, γ and λ are index parameters for each variable and μ is the random error term. STIRPAT is a nonlinear model containing multiple independent variables, and can therefore be converted to logarithmic form:

(2) lnI=lnα+βlnP+γlnA+λlnT+lnμ

where β, γ, and λ can be seen as the elasticity coefficients, which means that every 1% change in lnP, lnA, or lnT would lead to a β%, γ%, or λ% change in ln*I*. 

#### 2.2.2. Model Specification

Based on the STIRPAT model, our econometric model adopts a reduced form analysis to estimate the coefficients that reveal the relationships between population urbanization and PM_2.5_. The classical EKC hypothesis posits that environmental quality tends to at first deteriorate and then improve in line with urbanization in an inverted U-shaped curve. However, existing studies have shown that there may be U, N, and inverted N-shaped curves between environmental variables and urbanization. Therefore, the primary, quadratic, and cubic terms are decomposed to analyze and verify the nonlinear relationship between PM_2.5_ and population urbanization [37,38]. The model can be specified as follows

(3) $$\begin{array}{ll} \ln PM_{2.5i,t} & =\beta_{0}+\beta_{1}\ln Urban_{i,t}+\beta_{2}\ln Urban_{i,t}{}^{2}+\beta_{3}\ln Urban_{i,t}{}^{3}+\beta_{4}\ln P_{i,t}\\ & +\beta_{5}\ln A_{i,t}+\beta_{6}\ln T{\mathrm{ech}}_{i,t}+\beta_{7}\ln IS_{i,t}+\varepsilon_{i,t} \end{array}$$

where cities are denoted by the subscript i (i=1,…,N) and the subscript t (t=1,…,N) denotes the time period. $\beta_{0}$ is the intercept term for all individuals. $\beta_{1}$ to $\beta_{7}$ represent coefficients of the corresponding variable and $\varepsilon_{i,t}$ represents the random error term. 

$PM_{2.5}$ is our dependent variable. A series of annual average grids (2006–2016) of PM_2.5_ were obtained from the Battelle Memorial Institute and the Center for International Earth Science Information Network at Columbia University. The Global Annual PM_2.5_ Grids from MODIS, MISR, and SeaWiFS AOD with geographically weighted regression for the time period of 1998 to 2016 consist of annual concentrations (micrograms per cubic meter) of PM_2.5_, with dust and sea-salt removed [39]. Data for the YREB region were extracted from the global dataset and transformed into the GCS_WGS_1984 system using ArcGIS software (Esri, Redlands, CA, USA).

Population urbanization (lnUrban) is our explanatory variable. Since urbanization refers to the process of transforming agricultural population into urban population, the percentage of urban population relative to the total population can be used to represent city’s urbanization level. The population urbanization data were mainly collected from the statistical yearbooks of the nine provinces and two municipalities provided in Section 2.1. Additional data for several cities that are not available from this source were gathered from The Statistics Communique on National Economy and Social Development.

The other four control variables are as follows.

*P* represents population density. Considering the difference in administrative division areas between the cities, it is more appropriate to apply population density (population per unit area) to represent the impact of population concentration on PM_2.5_, rather than applying the total population [15].

*A* refers to the GDP per capita, which is a commonly used indicator representing urban economic development [26]. 

*T* is divided into two parts: technological progress Tech and industrial structure IS. First, technological progress is the main indicator for knowledge and ability and is also an important factor in controlling environmental pollution and alleviating PM_2.5_. We use the proportion of financial expenditure for science and education to denote this factor [40]. Second, fossil fuel combustion in secondary industries—including mining, manufacturing, electricity supply, and construction—may have an impact on air pollution [6]. Therefore, the proportion of secondary industry in the GDP is commonly used to represent the influence of industrial structure [41]. 

The data concerning these control variables were collected from Chinese Urban Statistical Yearbook [42]. Table 1 shows the statistical descriptions for all the variables.

#### 2.2.3. Dynamic Spatial Econometric Model

Many existing empirical analyses show that environmental pollution often shows certain path dependence characteristics. Therefore, it is of great importance to investigate the time-lag effect of PM_2.5_ changes [21]. The lagged item of PM_2.5_ is introduced into the STIRPAT model, by considering the dynamic cumulative effect of PM_2.5_.

(4) $$\ln PM_{2.5}=\alpha_{i,t}+\tau \ln PM_{2.5}{}_{i,t-1}+\beta_{1}\ln Urban_{i,t}+\beta_{2}{\left(\ln Urban_{i,t}\right)}^{2}+\beta_{3}{\left(\ln Urban_{i,t}\right)}^{3}+\beta_{4}X_{i,t}+\varepsilon_{i,t}$$

where $\varepsilon_{it}$ is the error term, i represents the different regions, and t indicates time. X represents a vector of the control variables.

Besides the dynamic cumulative effect, PM_2.5_ may have inevitable spatial autocorrelation and may be influenced by the consequent spatial spillover effects. That is, PM_2.5_ of city i may also be affected by its surrounding areas. The specification can be seen in the following equation.

(5) $$\ln PM_{2.5}{}_{i,t}=\delta W*PM_{2.5i,t}+\beta_{1}\ln Urban_{i,t}+\beta_{2}{\left(\ln Urban_{i,t}\right)}^{2}+\beta_{3}{\left(\ln Urban_{i,t}\right)}^{3}+\beta_{4}X_{i,t}+v_{t}$$

where W denotes the spatial weight matrix. In this analysis we use the distance between cities to establish the geographical weight matrix ($W_{ij}=1/d_{ij}^{2}$), which is obtained from the China Geographic Database of National Bureau of Measurement. δ, as the coefficient of $W*PM_{2.5}$, represents the spatial dependence of the sample observation.$v_{t}$ is a normally distributed disturbance term with a diagonal covariance matrix.

To perform a comprehensive analysis of the time-lag effect and spatial interaction effect, we adopt a dynamic spatial panel model, which controls the interference of the above factors to model estimation. This helps us avoid any estimation bias [43]. Combining Equations (4) and (5), the dynamic spatial panel model is set as follows

(6) $$\ln PM_{2.5}{}_{i,t}=\tau \ln PM_{2.5}{}_{i,t-1}+\delta W*\ln PM_{2.5i,t}+\beta_{1}\ln Urban_{i,t}+\beta_{2}{\left(\ln Urban_{i,t}\right)}^{2}+\beta_{3}{\left(\ln Urban_{i,t}\right)}^{3}+\beta_{4}X_{i,t}+\varepsilon_{i,t}+v_{t}$$

where τ represents the first-order lag regression coefficient of PM_2.5_ that reflects the influence of previous related factors on this period. δ represents the spatial lag regression coefficient, which in turn reflects the spatial spillover effect of air pollution.

## 3. Results

### 3.1. Spatial Distribution of PM_2.5_

**Figure 2:**
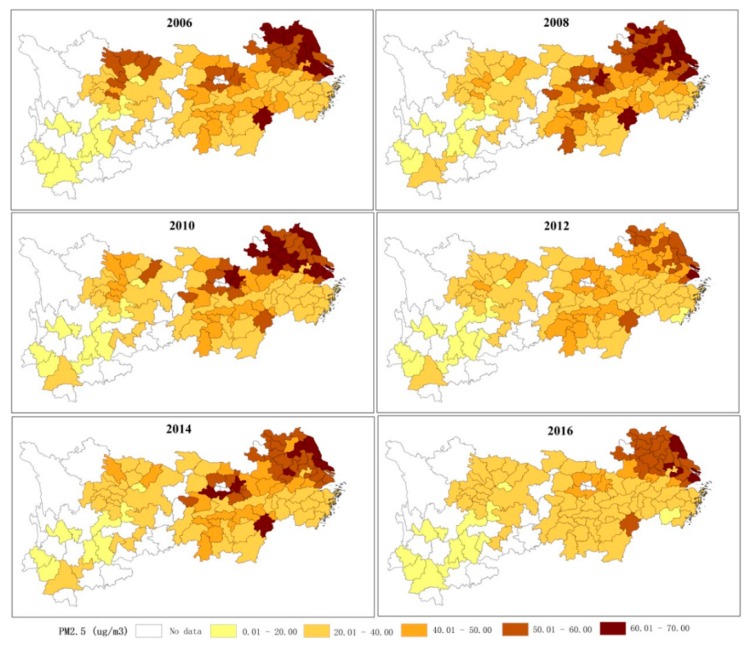
Spatial distribution evolution of PM_2.5_ in Yangtze River Economic Belt (YREB). The six figures from the upper left corner to the lower right corner represent the spatial distribution of PM_2.5_ in 2006, 2008, 2010, 2012, 2014, and 2016, respectively.

Figure 2 describes the spatial evolution pattern of the annual PM_2.5_ concentration in the study area for each city in the YREB in 2006, 2008, 2010, 2012, 2014, and 2016. The grid PM_2.5_ concentration data set combined AOD retrievals from multiple satellite instruments was provided by Aaron Van Donkelaar Professor of the Department of Physics and Atmospheric Science, Dalhousie University in Canada [38]. According to the natural fracture point classification, five concentration levels are established, ranging from high to low: low concentration level (2–20 μg/m^3^), medium–low concentration level (20–40 μg/m^3^), medium concentration level (40–50 μg/m^3^), medium–high concentration level (50–60 μg/m^3^), and high concentration level (60–70 μg/m^3^) [36]. 

The PM_2.5_ gradually increases along the Yangtze River from upstream to downstream (Figure 2). The spatial pattern of PM_2.5_ in YREB shows obvious spatial agglomeration. For example, the high concentration level is concentrated in the downstream area of the Yangtze River, especially in Shanghai and Jiangsu Provinces. PM_2.5_ in the upstream area of the YREB is lower than that in the downstream area. Generally, the number of hot spots shows an increasing trend during 2006, 2008, and 2010. That is, PM_2.5_ in YREB continues to deteriorate. Then, cities in the high concentration level of PM_2.5_ show a downward trend in the downstream area. Based on the spatial distribution of PM_2.5_, the regional and the temporal differences of PM_2.5_ pollution were found to be significant [44]. Therefore, it is of great importance to understand the regional differences when considering possible trends in the impact of urbanization on PM_2.5_ in different regions. Additionally, the economic and the overall urbanization level of the Yangtze River Delta all take precedence over the other areas in the YREB. Simultaneously, high urbanization cities, such as Shanghai, are currently experiencing high PM_2.5_ caused by population agglomeration. We therefore consider urbanization levels to explore the effect of different urbanization levels on PM_2.5_.

### 3.2. Spatial Autocorrelation Test

As described above, there might be spatial correlations in PM_2.5_ concentrations among adjacent cities [20]. We adopted the Global *Moran’s I* index in order to test the spatial autocorrelation of PM_2.5_ [45,46]. This commonly used index helps to measure the degree of overall spatial autocorrelation of the variables. It can be expressed as follows

(7) $$I=\frac{n\sum_{i=1}^{n}\sum_{j=1}^{n}\omega_{ij}(x_{i}-\bar{x})(x_{j}-\bar{x})}{\sum_{i=1}^{n}\sum_{j=1}^{n}\omega_{ij}\sum_{i=1}^{n}{\left(x_{i}-\bar{x}\right)}^{2}}$$

where n is the number of cities, which equals 108 in this study. $x_{i}$ and $x_{j}$ are the values of PM_2.5_ at location i and location j, respectively; $\bar{x}$ is the mean of PM_2.5_ and $\omega_{ij}$ is the element of the spatial weight matrix. The global *Moran’s I* value range from −1 to 1. A larger value of indicates a stronger spatial connection, while a smaller value indicates weaker spatial connection; if the *Moran’s I* value equals zero, then random spatial distributions exists, indicating no spatial correlation.

Table 2 presents the global *Moran’s I* statistics from 2006 to 2016. All values are positive at reasonable significance levels, indicating a positive spatial interdependence in PM_2.5_ in the YREB. Furthermore, cities with similar PM_2.5_ tend to be concentrated geographically. Over time, the overall trend of spatial integration of PM_2.5_ is intensified as the global *Moran’s I* value increased from 0.609 to 0.743. 

**Figure 3:**
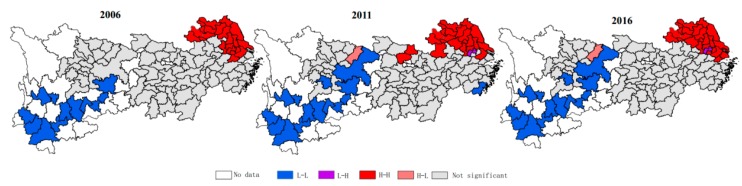
Scatter plot of *Moran’s I* values and LISA cluster map for PM_2.5_ in the YREB.

To further distinguish the spatial agglomeration patterns, a Moran scatter plot was used to test the average PM_2.5_ in the selected years. The Moran scatter plot and the Local Indicators of Spatial Association (LISA) cluster map of PM_2.5_ at the 0.05 significance level are presented in Figure 3. According to the analysis results, the sample areas can be divided into four agglomeration pattern types. The first quadrant is the H-H aggregation type, which indicates that the highly polluted areas are adjacent to each other. H-H regions mainly encompass the city of Shanghai and several cities in the Jiangsu, Zhejiang, and Anhui provinces in the downstream area of the Yangtze River. Similarly, L-L agglomeration regions in the third quadrant are concentrated in the upstream area and include the Sichuan and Yunnan provinces, as well as Chongqing. These regions tend to exhibit relatively lower levels of industrial development and low economic outputs per unit of construction [47]. Observably, regions in the L-L type agglomeration show an increasing tendency, extending from the upstream to midstream areas. The cluster map also shows that the range of H-L and L-H aggregations have not changed significantly from 2006 to 2016.

### 3.3. Full Sample Results

The estimation results employing different model specifications are shown in Table 3. 

The second column shows the results of Equation (3). Before estimating a panel data model, a fixed effect model or random effect model should be chosen using the Hausman test [48]. The result of the Hausman test implies that it is reasonable to employ a fixed effect model. Based on this model, the coefficients of lnUrban, ${\left(\ln Urban\right)}^{2}$ and ${\left(\ln Urban\right)}^{3}$ are 3.992, −1.137, and 0.104, respectively. All the coefficients are insignificantly positive at the 10% level across the second column, which indicates that there is not obvious relationship exists between urbanization and PM_2.5_ without considering spatial correlation and the time lag effect of the dependent variable. 

With the time-lag effect of the independent variable included, the system GMM result in the third column shows a strong time-lag effect for PM_2.5_. The estimated coefficient of $W*\ln PM_{2.5}{}_{i,t}$ is significantly positive (*p* < 0.01), that is, a high PM_2.5_ in the current period indicates a probability of the next period’s PM_2.5_ to continue increasing, pointing to an obvious lag effect. The global Moran value and local scatter plot show that PM_2.5_ among cities in the YREB has a stable positive spatial correlation, confirming the suitability of the spatial econometric model. Column 4 in Table 3 shows the spatial panel model result with spatial interaction effects based on Equation (5). The coefficient of $W*\ln PM_{2.5}{}_{i,t}$ is positive at the 1% significance test, which shows that there are significant spatial spillover effects for PM_2.5_. This means that a local PM_2.5_ change affects geographically neighboring regions. The result serves as a reminder that governmental efforts toward reducing environmental pollution should take a global view. As Table 3 shows, the lag effect of PM_2.5_ and the spatial interaction effect both exist. The urbanization effect on PM_2.5_ shown in columns 3 and 4 appears to be insignificant.

Most importantly, considering the spatial correlation and time-lag effect of PM_2.5_, next we implemented the dynamic spatial panel model. There are two important tests pertaining dynamic spatial panel data. First, the Sargan statistic of overidentification test is used to examine effectiveness and feasibility of instrumental variables. If the Sargan value fails to pass the significance test, the instrumental variables can be regarded as reliable. Second, the Arellano–Bond estimator is used to test the existence of sequence-dependent errors [49,50]. If the *p*-values of AR(2) are higher than 0.05, the disturbance term of the dynamic model does not exist with respect to the problem of sequence correlation. All the tests confirm that using a dynamic spatial model to detect the relationship between urbanization and PM_2.5_ is the most suitable. The estimated results of the dynamic spatial model can be seen in column 5 of Table 3, where the coefficients of $\ln PM_{2.5}{}_{i,t-1}$ and $W*\ln PM_{2.5}{}_{i,t}$ are 0.356 and 0.797, respectively, and statistically significant at the 1% level. Compared with the results of the system GMM and the spatial panel model, a difference occurs in the coefficients of lnUrban, ${\left(\ln Urban\right)}^{2}$, and ${\left(\ln Urban\right)}^{3}$. In the system GMM model, the primary, quadratic, and cubic terms of lnUrban are significant at the 10% level, while there is not an obvious association between population urbanization and PM_2.5_ in the spatial panel model. However, in dynamic spatial model, population urbanization has an obvious N-shaped effect on PM_2.5_ with two inflection points: 27% and 70%, respectively. Thus PM_2.5_ will continue to decrease as population urbanization increases from 27% to 70% during the second stage of the N-shaped curve. The result can be explained as the improvement of technology and sufficient capital conducted by government, which helps to control PM_2.5_ [21]. Additionally, people require healthier living conditions nowadays than before. Thereby PM_2.5_ begins to decrease with the agglomeration of population. 

### 3.4. The Heterogeneous Effects of Upstream, Midstream, and Downstream Cities

**Figure 4:**
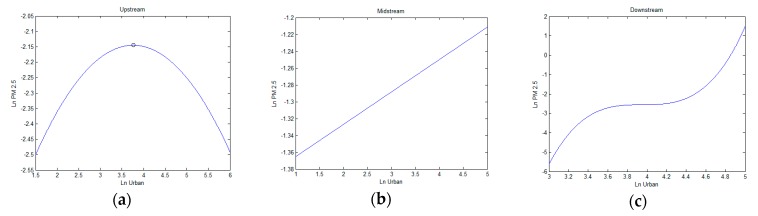
Diagrams relationships between population urbanization and PM_2.5_ in three areas: (**a**) upstream, (**b**) midstream, and (**c**) downstream.

The YREB is a vast area that is affected by numerous different natural, social, and economic. It thus displays significant diversity in urbanization and PM_2.5_. Considering the long-existing socioeconomic gap and watershed division among areas in the YREB, we split the sample according to upstream (31 cities), midstream (52 cities), and downstream (25 cities) areas to account for potential regional heterogeneities. The regression results for upstream, midstream, and downstream cities are shown in Table 4. Figure 4 shows the relationship trajectory between lnUrban and $\ln PM_{2.5}$ in the different regions.

In both upstream and downstream areas, regressions pass the AR(2) test for serial correlation and the Sargan test for overidentification. In the upstream area, the coefficients of the primary term of urbanization are significantly positive, while the coefficients of the squared term are negative. That is population urbanization shows an inverted U-shaped effect on PM_2.5_. Accordingly, the inflection point shown in Figure 4a is 43%, which demonstrates that urbanization increases PM_2.5_ below the inflection point, while increasing population urbanization restrains PM_2.5_ when goes over the 43% level. In the midstream area, when we add the primary term, the quadratic term, and the cubic term of to the model, the *p*-value of the AR(2) test is less than 0.05. That is, the model rejects the null hypothesis and that the two-order autocorrelation coefficient of the disturbance term is not different. Therefore, we dropped ${\left(\ln Urban\right)}^{2}$ and ${\left(\ln Urban\right)}^{3}$, and found that the coefficient of in this region is 0.039, indicating that a 1% increase in lnUrban would lead to a decrease in $\ln PM_{2.5}$ of 3.9% for the midstream region. A linear relationship exists between PM_2.5_ and urbanization, which can be seen in Figure 4b. In the downstream area, the coefficients of the lnUrban and ${\left(\ln Urban\right)}^{3}$ terms of population urbanization are both positive and the coefficient of ${\left(\ln Urban\right)}^{2}$ is negative. Thus, the relationship between PM_2.5_ and population urbanization is a monotonically increasing function in Figure 4c. This is similar to the full sample result. That is, population urbanization will promote PM_2.5_ in the downstream area. In sum, the increase of the population urbanization rate in the downstream and midstream regions will accelerate PM_2.5_ in the future [51]. Furthermore, the coefficient of the lagged dependent variable ($\ln PM_{2.5t-1}$) for the upstream area is significantly greater than that of the midstream and downstream areas. This implies that the lag-effect of air quality in the upstream region is more significant than in other areas.

The heterogeneity results can be explained from the perspectives of geographical conditions and the current development of the YREB region. First, the downstream and midstream regions have a relatively higher living standard and industrialization than the upstream region, which leads to a large population transfer, not only from local agricultural sectors, but also due to interregional migration [52]. Because of increasing population urbanization, as well as an increase in urban employment density, city sprawl is accompanied with an increase in building height and density, which is not beneficial to the rapid dispersion of PM_2.5_ concentration. Additionally, the population and labor force increase leads to a related increase in the number of vehicles, as well as energy consumption and infrastructure construction. All these factors will directly affect PM_2.5_ concentration. Secondly, the decrease of PM_2.5_ with the increase of population urbanization in the upstream area can be explained by the economic development and national policy. Economic development of upstream areas in the YREB are relatively lagging behind midstream and downstream, which lead to serious labor outflow and slow development of secondary industry. Furthermore, the national policy of “protecting the Yangtze River” prevents the upstream areas from investing in highly polluting industries. Over time more and more cities’ urbanization levels are higher than the inflection point of 43%, which reduces the impact of PM_2.5_.

### 3.5. The Heterogeneous Effects of Cities on Different Urbanization Levels

The PM_2.5_ effect of urbanization might also differ according to cities’ urbanization levels. This needs to be investigated further. To accomplish this, we divide the panel data of 108 cities into three groups according to the average urbanization level division for the study period of 2006 to 2016. Specifically, (1) the regions with an average urbanization ratio of 29.00% to 40.00% belong to low urbanization group; (2) the regions with average urbanization ratio of 40.01% to 50.00% belong to medium urbanization group; (3) and the regions with average urbanization ratio of 50.01% to 90% belong to high urbanization group.

**Figure 5:**
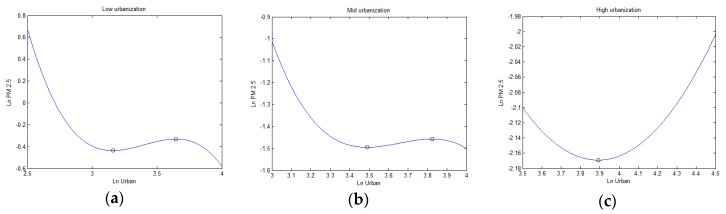
Diagrams relationship between population urbanization and PM_2.5_ at different urbanization levels: (**a**) low urbanization level, (**b**) medium urbanization level, (**c**) high urbanization level.

The results on the heterogeneous effects according to different urbanization levels are listed in Table 5. Figure 5 shows a diagram representing the relationship between lnUrban and $\ln PM_{2.5}$ for different urbanization levels. In columns 2 and 3, the coefficient of lnUrban and ${\left(\ln Urban\right)}^{3}$ are negative, the term of ${\left(\ln Urban\right)}^{2}$ is positive, and the *p*-values are all less than 5%. Therefore, an inverted N-shaped relationship exists in the low and medium urbanization level groups in Figure 5a,b. For the cities in the low urbanization level group, the first inflection point is 24%, and the second is 38%. For the medium urbanization level group, the two inflection points are 33% and 46% respectively. To further explore the dynamic impacts of urbanization on PM_2.5_, we calculate the number of cities located in the different stages of the three curves. Considering the urbanization ratio, the two groups are nearly between the two inflection points and pass over the second inflection points. Therefore, increased urbanization first raised PM_2.5_ in regions with low urbanization and medium urbanization and then reduced the PM_2.5_.

In column 4, the coefficients of the primary, squared, and cubic terms of urbanization are statistically insignificant. We therefore excluded ${\left(\ln Urban\right)}^{3}$ and reestimated the model (column 5). Judging from the coefficients in column 5, a U-shaped curve exists in the high urbanization group, with an inflection point of 49% in Figure 5c. Generally, the urbanization growth would first reduce PM_2.5_ in this region and then increase the PM_2.5_ again. Table 6 shows that all the cities in the high urbanization level group have passed over the inflection point of the U-shaped curve since 2010, which indicates that the PM_2.5_ is aggravated with the increasing urban population.

Section 3.5 (listed above) confirms that for cities with low or medium urbanization level, population urbanization showed a positive impact on PM_2.5_ in the past. However, due to the rapid growth of urbanization, population urbanization has passed the second inflection point and will have a long-term negative effect on PM_2.5_. These cities are mostly remote cities with relatively low overall urbanization levels, which will not promote PM_2.5_ because of their safe and comfortable development mode. For cities with a high urbanization level, population urbanization has surpassed the inflection point of the U-shape curve and will maintain a positive impact on PM_2.5_ for a long time. Many cities in the high urbanization group such as Shanghai, Nanjing, Hangzhou, Wuxi, Suzhou, and other developed areas are eager to pursue economic and industrial development, which will inevitably lead to a long-term increase in population urbanization. This is similar to the results of the downstream area of YREB. This pursuit of development of these regions—that have already reached a certain urbanization level—will lead to a further continuous increase of population urbanization. With the urban population extremely inflated, these cities will face increased traffic congestion, accelerated real estate construction, and industrial development caused by population agglomeration, and PM_2.5_ will eventually increase.

## 4. Discussion

Despite the rising interest in understanding the impact of urbanization on air quality, few empirical studies have been able to fully explain this impact. This is partly due to the complicated nature of statistical analysis in such a case, given the presence of factors such as heterogeneous effects, dynamic effects, and spatial dependence. Our study aims to fill this gap in existing research by using a more comprehensive econometric model and focusing on the heterogeneous effect. Considering the time-lag effect and spatial interaction of PM_2.5_, the study used a STIRPAT model to analyze the nonlinear relationship between population urbanization and PM_2.5_ based on panel data of 108 prefecture-level cities in the YREB from 2006 to 2016. The results indicate that the relationship shows an N-shaped curve for the YREB. The traditional EKC inverted U curve is not applicable to PM_2.5_ and population urbanization in different models, which indicates that population urbanization has obvious stage heterogeneity from the perspective of regional heterogeneity and urbanization level heterogeneity on PM_2.5_.

Based on the above statements concerning regional heterogeneity and urbanization level heterogeneity, we find that (1) for cities in the downstream and midstream areas, as well as those with high urbanization levels, there was a significantly positive trend proving that urbanization will increase PM_2.5_. More attention should be paid to the rapid economic development of cities that have exceeded the inflection point or will soon exceed the urbanization inflection point of 49%, and thus have a positive impact on PM_2.5_. (2) Regarding the upstream areas of the YREB and the small and medium-sized cities with low urbanization level: although urbanization has passed through the increasing stage in relation to PM_2.5_, these areas have passed the second inflection point of the N-shaped curve and have begun to reduce PM_2.5_. Thus, when addressing PM_2.5_, the government cannot simply blame air pollution purely on factors related to population agglomeration. For cities with different urbanization levels, the impact of future urbanization development on PM_2.5_ also differs. Therefore, solutions should be found that suit specific circumstances, especially for the areas with a low level of economic development, the source of PM_2.5_ should be determined and controlled suitably.

## 5. Conclusions

In conclusion, PM_2.5_ remains a serious environmental challenge in the YREB, especially in most downstream and midstream regions. Considering the entire YREB, PM_2.5_ showed a trend of firstly decreasing and then increasing with the increasing of urbanization from 2006 to 2016. In the upstream area, population urbanization shows an inverted U-shaped effect on PM_2.5_, while presenting an increasingly linear relationship with midstream and downstream. From the perspective of urbanization levels, a similar inverted N-shaped curve exists in the medium and low urbanization level groups, and a U-shaped relationship exists in the high level of urbanization.

## Figures and Tables

**Table 1:** Summary statistics.

Variable	Type of Variables	Units of Measurement	Mean	Standard Deviation	Min	Max
PM_2.5_	Dependent variable	μg/m^3^	40.15	14.883	2.41	74.04
*Urban*	Explanatory variable	%	48.45	13.535	14.58	91.24
P	Control variable	person/km^2^	898.58	632.360	53.16	3934.35
A	Control variable	Yuan	49,110.74	34,216.63	4490	43,9321
Tech	Control variable	%	0.17	0.05	0.05	0.36
IS	Control variable	%	50.55	10.50	15.89	79.14

**Table 2:** Global *Moran’s I* results.

Year	*Moran’s I*	Standard Deviation	*p*-Value
2006	0.609	0.0624	0.001
2007	0.721	0.0631	0.001
2008	0.698	0.0651	0.001
2009	0.706	0.0617	0.001
2010	0.657	0.0664	0.001
2011	0.661	0.0637	0.001
2012	0.622	0.0644	0.001
2013	0.663	0.0658	0.001
2014	0.670	0.0640	0.001
2015	0.778	0.0635	0.001
2016	0.743	0.0627	0.001

**Table 3:** Regression results of full sample.

Determinants	Fixed Effect Model	System GMM	Spatial Panel Model	Dynamic Spatial Model
$\ln PM_{2.5i,t-1}$		0.798 ***		0.356 ***
		(0.029)		(0.012)
$W*\ln PM_{2.5}{}_{i,t}$			0.833 ***	0.797 ***
			(0.031)	(0.019)
lnUrban	3.992	−10.473 *	0.361	5.231 *
	(2.691)	(5.495)	(1.877)	(2.934)
${\left(\ln Urban\right)}^{2}$	−1.137	2.873 *	−0.124	−1.406 *
	(0.760)	(1.545)	(0.527)	(0.799)
${\left(\ln Urban\right)}^{3}$	0.104	−0.263 *	0.013	0.124 **
	(0.071)	(0.143)	(0.049)	(0.072)
lnP	0.010	0.098 **	−0.011	0.120 ***
	(0.015)	(0.042)	(0.010)	(0.019)
lnA	−0.087 ***	−0.024 ***	−0.018 *	0.013 **
	(0.012)	(0.021)	(0.010)	(0.007)
lnTech	−0.015	−0.017	−0.003	−0.019 *
	(0.014)	(0.034)	(0.010)	(0.012)
lnIS	0.151 ***	0.392 ***	0.054 *	0.053 ***
	(0.014)	(0.074)	(0.030)	(0.014)
Constant	−0.622	11.536*		−8.086 **
	(3.176)	(6.503)		(3.579)
Observations	1188	1080	1188	1080
R-squared	0.231		0.251	0.9456

Note: There are no inflection points, because the functions are monotonically decreasing. *, **, and *** represent significant differences at the 10%, 5%, and 1% levels, respectively. The dynamic spatial model passes the AR(2) test for serial correlation and the Sargan test for overidentification.

**Table 4:** Heterogeneity results by upstream, midstream, and downstream cities.

Variables	Regional Heterogeneity				
	Upstream	Midstream			Downstream
$\ln PM_{2.5i,t-1}$	0.545 ***	0.148 ***	0.149 ***	0.164 ***	0.146 ***
	(0.037)	(0.007)	(0.007)	(0.009)	(0.036)
$W*\ln PM_{2.5}{}_{i,t}$	0.996 ***	1.093 ***	1.097 ***	1.051 ***	1.273 ***
	(0.038)	(0.010)	(0.008)	(0.008)	(0.021)
lnUrban	0.522 **	0.130	0.289 ***	0.039 ***	164.259 ***
	(0.235)	(0.089)	(0.022)	(0.005)	(63.064)
${\left(\ln Urban\right)}^{2}$	−0.069 *	0.009	−0.023 ***		−41.521 ***
	(0.036)	(0.017)	(0.002)		(15.772)
${\left(\ln Urban\right)}^{3}$	0.003	−0.002 **			3.499 ***
	(0.002)	(0.001)			(1.313)
lnP	0.171 ***	−0.001	−0.001	−0.001	−0.011
	(0.028)	(0.001)	(0.001)	(0.002)	(0.007)
lnA	0.107 ***	0.006 ***	0.007 ***	0.009 ***	0.008
	(0.018)	(0.002)	(0.001)	(0.002)	(0.010)
lnTech	−0.024*	0.018 ***	0.019 ***	0.020 ***	−0.005
	(0.015)	(0.003)	(0.002)	(0.002)	(0.011)
lnIS	−0.493 ***	0.071 ***	0.064 ***	0.093 ***	0.221 ***
	(0.068)	(0.011)	(0.010)	(0.010)	(0.073)
Constant	−3.128 ***	−1.761 ***	−2.006 ***	−1.404 ***	−219.2 ***
	(0.564)	(0.162)	(0.068)	(0.076)	(83.73)
Observations	310	520	520	520	250
AR(1)	**0.000**	0.000	0.000	**0.000**	**0.000**
AR(2)	**0.558**	0.009	0.009	**0.316**	**0.079**
Sargan	1.000	0.870	0.832	0.873	1.000
Trajectory	Inversed U	—	—	Line	Line
Inflection point	43%				

Note: There are no inflection points, because the functions are monotonically decreasing. *, **, and *** represent significant differences at the 10%, 5%, and 1% levels respectively. Column 2, 5, and 6 pass the AR(2) test for serial correlation (marked in bold) and the Sargan test for overidentification.

**Table 5:** Heterogeneity results according to cities on different population urbanization levels.

Variables	Urbanization Heterogeneity			
	Low Urbanization Level	Medium Urbanization Level	High Urbanization Level	
$\ln PM_{2.5i,t-1}$	0.585 ***	0.412 ***	0.190 ***	0.189 ***
	(0.043)	(0.021)	(0.017)	(0.017)
$W*\ln PM_{2.5}{}_{i,t}$	0.535 ***	0.766 ***	1.191 ***	1.198 ***
	(0.080)	(0.025)	(0.021)	(0.023)
lnUrban	−63.703 ***	−81.670 ***	45.997	−3.488 **
	(21.886)	(26.471)	(32.127)	(1.570)
${\left(\ln Urban\right)}^{2}$	18.806 ***	22.383 ***	−11.637	0.448 **
	(6.454)	(7.133)	(7.915)	(0.194)
${\left(\ln Urban\right)}^{3}$	−1.842 ***	−2.040 ***	0.983	
	(0.631)	(0.640)	(0.650)	
lnP	0.0594 *	0.017 ***	0.004	0.001
	(0.033)	(0.006)	(0.006)	(0.006)
lnA	−0.021	0.017 ***	0.007 **	0.006 **
	(0.022)	(0.005)	(0.003)	(0.003)
lnTech	−0.021 *	0.013 **	0.016 ***	0.016 ***
	(0.011)	(0.0062)	(0.004)	(0.004)
lnIS	−0.050	0.115 ***	0.149 ***	0.147 ***
	(0.071)	(0.029)	(0.047)	(0.045)
Constant	71.151 ***	97.662 ***	−62.866	4.620
	(24.639)	(32.798)	(43.379)	(3.094)
Observations	320	350	410	410
Sargan test	1.000	0.999	0.995	0.996
AR(1)	0.001	0.000	0.000	0.000
AR(2)	0.352	0.707	0.846	0.854
Trajectory	Inverted N	Inverted N	—	U
Inflection point	24%, 38%	33%, 46%	—	49%

Note: There are no inflection points, because of the functions are monotonically decreased. *, **, and *** represent significant differences at the 10%, 5%, and 1% levels, respectively. Columns 2, 3, and 5 pass the AR(2) test for serial correlation and the Sargan test for overidentification.

**Table 6:** Number of cities located in different population urbanization levels.

Year	Low Urbanization Level			Medium Urbanization Level			High Urbanization Level	
	<24	24–38	>38	<33	33–46	>46	<49	>49
2006	7	25	0	11	24	0	17	24
2007	4	28	0	2	33	0	13	28
2008	1	31	0	0	34	1	5	36
2009	1	30	1	0	29	6	2	39
2010	1	29	2	0	26	9	0	41
2011	1	24	7	0	22	13	0	41
2012	0	18	14	0	16	19	0	41
2013	0	14	18	0	14	21	0	41
2014	0	10	22	0	8	27	0	41
2015	0	7	25	0	3	32	0	41
2016	0	4	28	0	0	35	0	41
